# 543 Diversity in Burn Surgery Programs

**DOI:** 10.1093/jbcr/irad045.140

**Published:** 2023-05-15

**Authors:** Samantha Delapena, Andy Chen, Abraham Houng, Rachelle Lodescar

**Affiliations:** New York Presbyterian - Weill Cornell Medicine, New York, New York; New York Presbyterian - Weill Cornell, New York, New York; Weill Cornell Medical College, New York, New York; New York Presbyterian, Elmsford, New York; New York Presbyterian - Weill Cornell Medicine, New York, New York; New York Presbyterian - Weill Cornell, New York, New York; Weill Cornell Medical College, New York, New York; New York Presbyterian, Elmsford, New York; New York Presbyterian - Weill Cornell Medicine, New York, New York; New York Presbyterian - Weill Cornell, New York, New York; Weill Cornell Medical College, New York, New York; New York Presbyterian, Elmsford, New York; New York Presbyterian - Weill Cornell Medicine, New York, New York; New York Presbyterian - Weill Cornell, New York, New York; Weill Cornell Medical College, New York, New York; New York Presbyterian, Elmsford, New York

## Abstract

**Introduction:**

Racial and gender disparities have been longstanding in the fields of medicine and surgery. In the literature, underrepresentation has been highlighted in plastic surgery and general surgery programs, however few studies examine this within burn surgery. Two studies describe the gender and racial disparities that exist in burn surgery leadership. This study seeks to compare and highlight the representation of minority and female surgeons in burn surgery training at a major metropolitan burn center to other surgical programs.

**Methods:**

Applicant data were reviewed for acceptance to the clinical burn surgery fellowship program from 2008 to 2022. Self-identification was used to categorize gender as male or female and race as Asian, African American, White, and other. The gender and race of each applicant was recorded. The data was analyzed and compared to data trends in female and minority representation among physicians in other training programs. Demographic data were also obtained from newly hired staff of the same metropolitan burn center.

**Results:**

From 2008-2022, there have been 30 physicians enrolled in this institution’s burn surgery fellowship program. Fourteen (46.7%) were female. Twelve (40%) were White, ten (33.3%) were African American, six (20%) were Asian, and two (6.7%) were of unknown racial background. From 2018-2022, there have been 277 newly hired staff members for the burn center. There were 238 females (85.9%). There were 142 (51.2%) who were White, 82 (29.6%) who were African American, 30 (10.8%) who were Asian, and 23 (8.3%) who were Hispanic or Latino.

**Conclusions:**

In 2022, the US graduating medical class was 52% female – a proportion that has slightly increased over the last four years. Furthermore, the US graduating medical school class was 58.8% White, 25.5% Asian, 10.6% Hispanic or Latino, 8.3% Black; these proportions of minority groups have also demonstrated a slow, but steady, increase over the last four years. Previous studies have demonstrated a lack of diversity among burn surgery leadership, but do not address diversity among burn surgery fellows or among newly recruited medical staff within burn centers. This institution’s burn surgical program has aimed to maintain a diverse minority representation to serve a community that is also diverse in race, gender, and socioeconomic status.

**Applicability of Research to Practice:**

Increasing gender and racial representation in burn surgery programs is beneficial for supporting the burn patient population, which is frequently diverse in race, gender, and socioeconomic status.

